# Visualizing Degradation of Black Phosphorus Using Liquid Crystals

**DOI:** 10.1038/s41598-018-31067-4

**Published:** 2018-08-28

**Authors:** Bilal Abbas Naqvi, Muhammad Arslan Shehzad, Janghwan Cha, Kyung-Ah Min, M. Farooq Khan, Sajjad Hussain, Yongho Seo, Suklyun Hong, Jonghwa Eom, Jongwan Jung

**Affiliations:** 1 Departement of Nanotechnology &Advanced Materials Engineering, Sejong University, Neungdong-Ro 209, Seoul, 05006 South Korea; 2 Graphene Research Institute, Sejong University, Neungdong-Ro 209, Seoul, 05006 South Korea; 3 Departement of Physics & Astronomy, Sejong University, Neungdong-Ro 209, Seoul, 05006 South Korea; 4 Present Address: School of Chemical and Biomolecular Engineering, Georgia Institute of Technology, Atlanata Georgia, 30318 USA

## Abstract

Black Phosphorus (BP) is an excellent material from the post graphene era due to its layer dependent band gap, high mobility and high I_on_/I_off_. However, its poor stability in ambient poses a great challenge for its practical and long-term usage. The optical visualization of the oxidized BP is the key and the foremost step for its successful passivation from the ambience. Here, we have conducted a systematic study of the oxidation of the BP and developed a technique to optically identify the oxidation of the BP using Liquid Crystal (LC). It is interesting to note that we found that the rapid oxidation of the thin layers of the BP makes them disappear and can be envisaged by using the alignment of the LC. The molecular dynamics simulations also proved the preferential alignment of the LC on the oxidized BP. We believe that this simple technique will be effective in passivation efforts of the BP, and will enable it for exploitation of its properties in the field of electronics.

## Introduction

The 2D layered materials, such as graphene and transition metal di-chalcogenides (TMDs), have opened a new era for electronic devices. In post graphene material, BP stands out among them due to its unique electronic and structural properties. It has strong in-plane anisotropy due to a puckered orthorhombic crystal lattice in which one phosphorus atom is bonded to three other phosphorus atoms. A monolayer of the BP, phosphorene has a direct band gap of 2 eV which decreases to 0.3 eV in bulk. The reason for this transition in band gap lies in the increase of the layer to layer interaction in the van der Waal structure^1,2^. The presence of the band gap and the high value of charge mobility makes BP a promising candidate for field effect transistors with effective switching^2–4^. Due to its unique electronic structure, BP has also shown excellent performance in optoelectronics and memory devices^5–7^. BP shows both a p and an n-type behaviour as well as ambipolar transport characteristics depending upon the contact metal used^3^.

However, due to the presence of the lone pairs with each phosphorus atom in the BP, it is strongly reactive and readily oxidized. Oxidation is one of the most crucial problems with BP. The surface of the relatively thin BP flakes, which are the most suitable candidates for electronic devices, easily get oxidized within an hour after cleavage from bulk if exposed to an ambient environment^1,8^. This oxidation causes irreparable damages to the physical characteristic of the BP^9–11^. It has been reported that the oxidation rate increases when the BP is exposed to light^12^. Walia *et al*. also reported that BP noticeably deteriorated when the BP was irradiated with a UV light for several minutes^13^. In this work, the AFM analysis confirms the oxidation of the BP as the surface roughness increased. Until this point, the oxidation is not limited to the surface, but it caused adverse effects, such as volumetric changes, imbalanced dipole introduction and a complex oxides species (P_x_O_y_) formation^1,12,14,15^. Furthermore, when exposed for a long time, the etching of BP starts and it becomes invisible due to constant thinning due to the oxidation^1^. Many methods have been reported for the passivation of the BP, such as encapsulating BP with hBN, growing stable phosphorus oxide using plasma, coating high-k oxides, and chemcial passivation that use ionic liquids and metal ion modification^16–18^. It has also been reported that the AFM, the Raman analysis and the TEM can be used to investigate the oxidation chatacteristics of the BP. Walia et.al. used the AFM to study the oxidation of the BP, and Hirsch *et al*. used the Raman mapping analysis to investigate the BP^13,14^. However all these techniques require a long time and a sophisticated exprimental setup. In addition to all these obstacles, oxidation of the BP is not visible under an optical microscope at an early stage until irreparable damage to the BP occurs and its physical appearance changes. Here we reported a simple method to optically visualize the oxidation of the BP using a polarized optical microscope and liquid crystal (LC). This technique can also be used to view the completely obselete BP and its remanents as P_x_O_y_ at an early stage.

In the past the defects on the 2D materials, such as the oxidation defects and the grain boundaries, are studied with LC alignment on them^19,20^. Defects, such as the oxidation defects and the pin holes on a single domain of graphene, were visualized using nematic LC^21^. LC has a unique ability to orient itself on different surfaces depending upon the weak physical interaction that arises due to the electrical/magnetic field, the surface modification and the presence of defects on the surface^22,23^. LC is an anisotropic material and exhibits optical birefringence. The 2D materials have a broken pi stacking, which favours the adsorption and the alignment of the LC on their surfaces. This alignment strongly depends upon the crystalline orientation. This alignment is disturbed due to any surface modifications or changes in the crystalline orientation. The nematic phase of the LC gained a lot of significance after its alignment has been studied on synthetically grown 2D materials, such as graphene, hBN and TMDs, for the optical visualization of their grains^19,24,25^. In the nematic phase, all the molecules in the LC are stacked parallel to an axis called the director. The birefringence property of the nematic LC helps to optically analyze the minute changes based on the alignment of the LC. This birefringent behaviour in the LC makes it a suitable option to study the anisotropy as well as the surface defects. The LC has been previously used to optically study the grain boundaries and the surface defects in the graphene and the TMDs^21^.

There are numerous ways of stabilizing black phosphorus, such as using a dielectric thin film, encapsulating the BP in hBN, and passivation using ionic liquids^11,17,18^. However, there aren’t any reports of a method to optically visualize the BP in an early stage. In this study, the nematic LC (5CB) was used to optically study the oxidation defects on few-layer BP flakes under a polarized optical microscopy (POM). A UV light was used to conduct an in-depth analysis of the oxidation by enhancing the oxidation of the BP. The LC alignment visualizes the oxidation defects generated on the surface. We believe this work not only visualizes the defects of the BP but also contributes to study the alignment behaviour of the LC molecules on the BP.

## Results and Discussion

**Figure 1:**
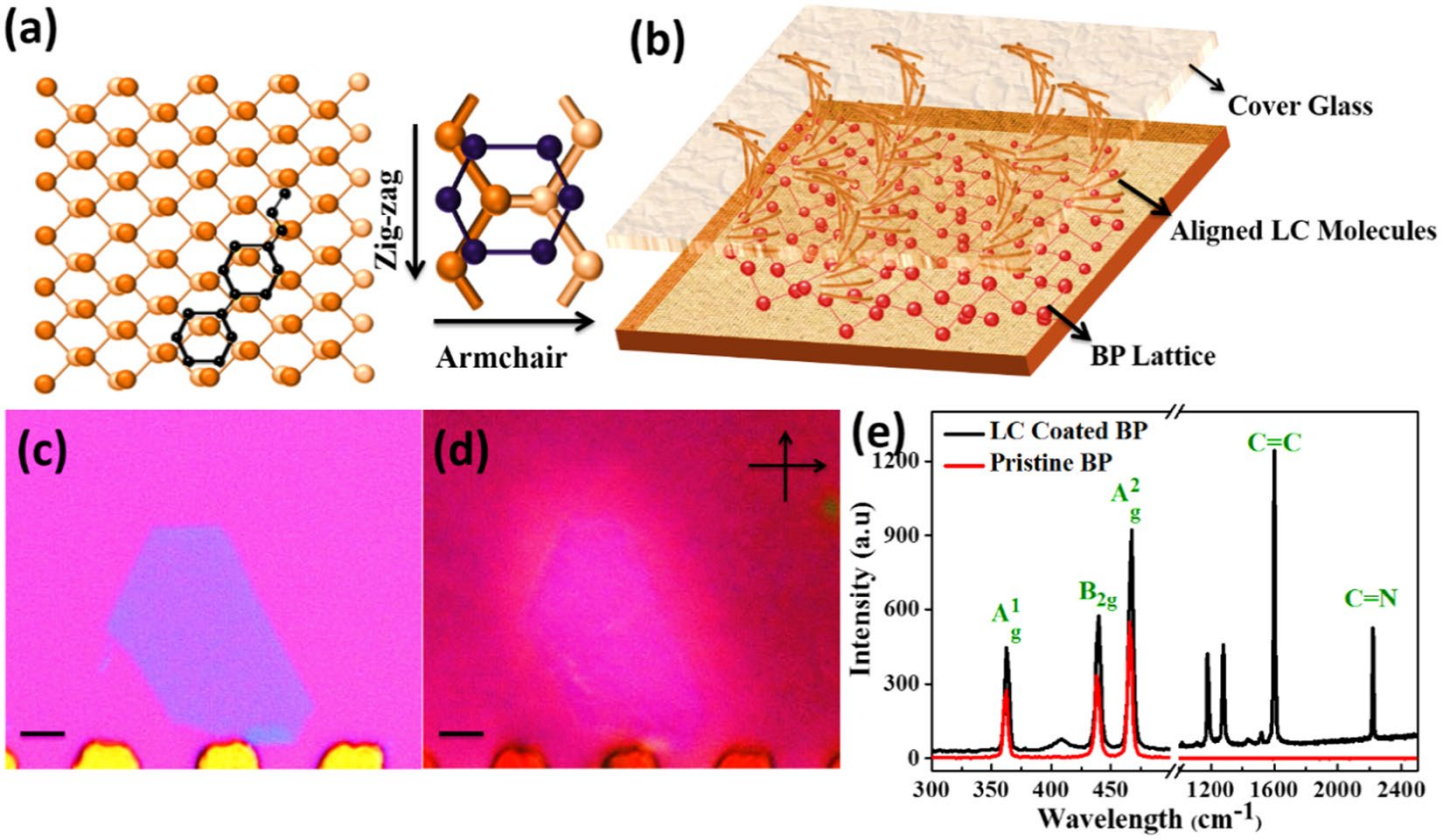
The LC alignment on the BP. (**a**) A schematic top view of the LC molecule on the BP lattice. (**b**) A schematic of the LC coated BP with a cover glass. (**c**) An optical image of the BP Flake. (**d**) A POM Image of the LC coated BP confirms the alignment of the LC (**e**) A Raman spectra of the Pristine BP and the LC coated BP. The scale bar = 10 μm.

BP was exfoliated from a bulk chunk using the micromechanical cleavage method, and then it was transferred using a PDMS stamp on a Si/SiO_2_ substrate which is illustrated in Fig. 1c. The gold pattern was fabricated to mark the coordinates of the flakes. The LC 5CB was coated on the sample using a spin coater. Polyvinyl Alcohol (PVA) was coated on a thin glass slide and then rubbed in a specific direction. The glass slide was placed on the sample so that the coated LC film and the PVA were in contact. These grooves in the thin PVA film helped to align the LC molecules in one direction. Using this method, only the alignment on the desired alignment film, (which is the BP in this case) can be studied independently. (Schematic is shown in Fig. 1b).

In the Fig. 1a,b, the schematic representation of the LC alignment on the BP flake is shown. Due to the broken pi stacking, the LC tended to align on the 2D materials. In the BP, there are two primary atomic orientations, which are the zigzag and the armchair at 90° with each other. With the density function theory calculation it turns out that the LC tends to align preferentially along the armchair orientation (Details regarding the density function theory are given in the calculations section). Figure 1c is the optical image of a few-layer BP flake, which was transferred on the Si/SiO_2_ substrate using the dry transfer technique. This flake was coated with the LC and observed under POM. In Fig. 1d, it was observed that the LC coated BP flakes exhibited a single color. A pristine BP flake was exfoliated from a single crystal and had a single crystalline orientation throughout. Therefore, the LC was uniformly aligned on this flake, and this flake appeared bright when viewed with a POM. In Fig. 1d. the flake appeared brighter which means that the director axis of the aligned LC is in between the perpendicularly cross analyzer and the polarizer. Figure 1e is the Raman spectra of the BP with and without an LC coating. Three peaks, which are A_g_^1^, B_2g_ and A_g_^2^, are present in the Raman spectra of the pristine BP. While in LC coated BP apart from the three signature peaks of the BP, the peaks of C=C, C=N, and the biphenyl stretching peaks from the LC were also present.

As discussed earlier, a few atomic layers of thick BP is unstable in ambient conditions. The BP has a strong puckered structure in which the phosphorus (P) atoms are covalently bonded to the 3 other phosphorus atoms. Each P atom has a lone pair, which makes the BP highly reactive in ambient^15^. Although this surface oxidation doesn’t cause the any lattice distortion, however, the two strong dipoles are created rendering the BP hydrophilic due to the formation of P-O bonds. This hydrophilicity is the further cause of a more complex defect formation. This may cause volumetric changes in the lattice and eventually lead to a complete loss of the BP due to the formation of the complex oxides and the acidic species^11,15^.

**Figure 2:**
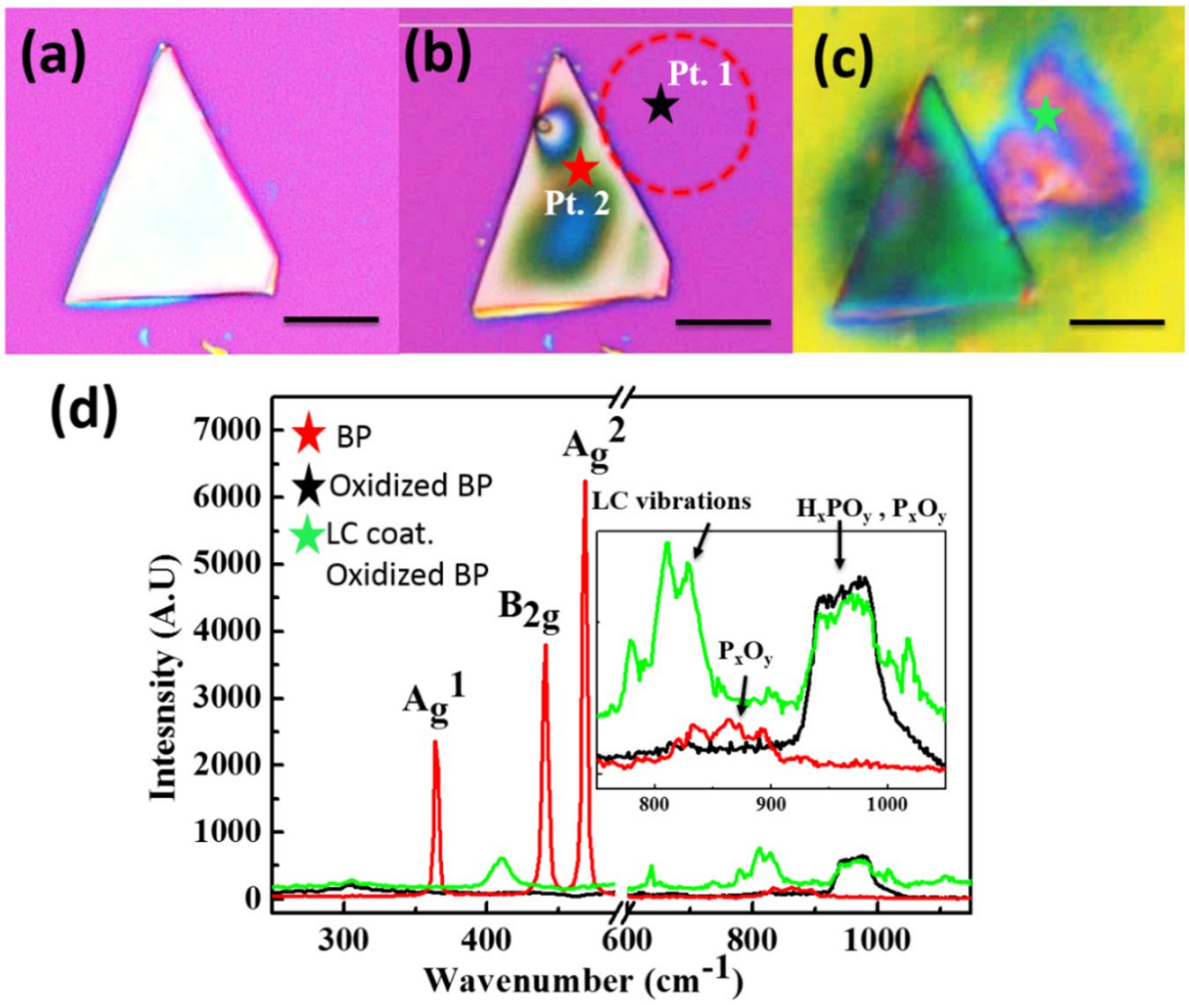
The LC alignment on the oxidized BP due to the ambient exposure and the Raman analysis. (**a**) The freshly cleaved BP flake. (**b**) The oxidized BP flake which was exposed to the ambient and the oxidation is visible. (**c**) The LC coated BP in which the oxidation remnants of a thin heavily oxidized flake are visible next to a triangular flake. (**d**) The Raman Spectra at three points indicated by the stars. The red star indicates the Raman taken on the BP flake while the black and the green stars indicate the Raman at completely oxidized region with and without the LC. The BP flakes show three sharp signature characteristic peaks, which indicate a bulk flake. A small broad peak at 800–900 cm^−1^ on the BP corresponds to the mixed phosphorus oxides vibrations. At the completely oxidized regions which are indicated by the red and the green stars, there is a relatively intense peak at 900–1000 cm^−1^. This corresponds to the mix signals from the phosphoric acids and the phosphorus oxides species. The offset is zoomed for the peaks that emerged in the range of 750 to 1100 cm^−1^.

To further extend the analysis of the oxidation of the BP and the alignment behaviour of LC on the BP, a triangularly shaped flake, shown in Fig. 2a was exposed to the atmosphere to get oxidized. This flake was a relatively thicker flake, and keeping this in view it was exposed to ambient conditions for 48 hours. It gets oxidized consequently, which is evident in the Fig. 2b. This flake was then coated with the LC and observed under a POM. The interference segments observed on this flake indicated that the LC has several alignments on this flake. These different alignments indicate a non-uniformity, which evidently proved the presence of the oxidation defects on BP, which is shown in Fig. 2c. When the sample was coated with the LC and observed under a POM, interestingly we found remnants of a completely oxidized BP at a point which is indicated as Pt. 1 in Fig. 2b, which was invisible otherwise. The LC molecule aligns on these remnant oxides due to a weak physical interaction. Due to the birefringence of the aligned LC molecules, this heavily oxidized flake can be seen. However, it is worth noting that in the absence of LC there aren’t any visual signs of the presence of these residual oxides at Pt1. Thus, the LC serves as a powerful and a simple ocular tool to visualize the obsolete BP and its remnants after oxidation.

The Raman analysis was conducted at Pt. 1 and Pt. 2 in order to confirm the nature of these residual oxides, as shown in Fig. 2d. At Pt. 2, the three signature peaks of BP A^1^_g_, B_2g_ and A_g_^2^ are present at 362 cm^−1^, 439 cm^−1^ and 468 cm^−1^ respectively. Apart from these three modes, the Raman analysis also showed a broad but prominent peak around 800–900 cm^−1^. This broad peak corresponds to the vibration modes of the P_x_O_y_^26,27^. At the Pt. 1, the Raman analysis didn’t provide any evidence for the BP. However, a relatively strong and broad peak around 900–1000 cm^−1^ was present. This peak corresponds to the mixed signals from the vibrations of the phosphoric acids and also some of the vibrations of the phosphoric oxides^13,28^. The LC was coated on the sample and a Raman spectrum was also collected which is indicated by a green star at the oxidized region with the LC coating. However, we didn’t find any considerable shifts in the newly emerged peak, but there was only a small decrease in intensity. The emergence of the vibration modes from the phosphoric acids was due to the formation of the acidic species from the reaction between the oxides and the moisture present in the ambient. The formation of the acidic species led to the subsequent etching of the BP, which made it almost invisible. Another BP flake was cleaved and subjected to the O_2_ plasma treatment for a different perspective. After 15 sec of the plasma treatment the flakes disappeared when observed using the optical microscope. However, the LC coating made it visible under a POM which is illustrated in Fig. S3.

The 2D materials for example the MoS_2_, undergo an oxidational decay upon the UV irradiation^19,21,29^. The BP behaved differently under dark and illuminated environments^12^. The thinner flakes of the BP were more prone to oxidation and had quicker oxidation kinetics. According to previous reports, the redox potential of O_2_/O_2_^−^ lies in the band gap of monolayer phosphorene^30^. Excitons generated from the conduction band of the BP radicalized the O_2_ which reacted with the BP. However, with an increase in the number of layers, the band gaps increased for the bulk and it eventually fell below the redox potential of the O_2_/O_2_^−^ making the oxidation difficult for the bulk BP^30^. The UV light can make oxidation thermodynamically favourable for the bulk BP by exciting excitons from the conduction band. Also, it can increase the rate of oxidation for the thinner BP whose oxidation was already favourable without any UV exposure.

$${{\rm{O}}}_{2}+\hbar {\rm{v}}\to {{{\rm{O}}}_{2}}^{-}+{\rm{h}}$$

**Figure 3:**
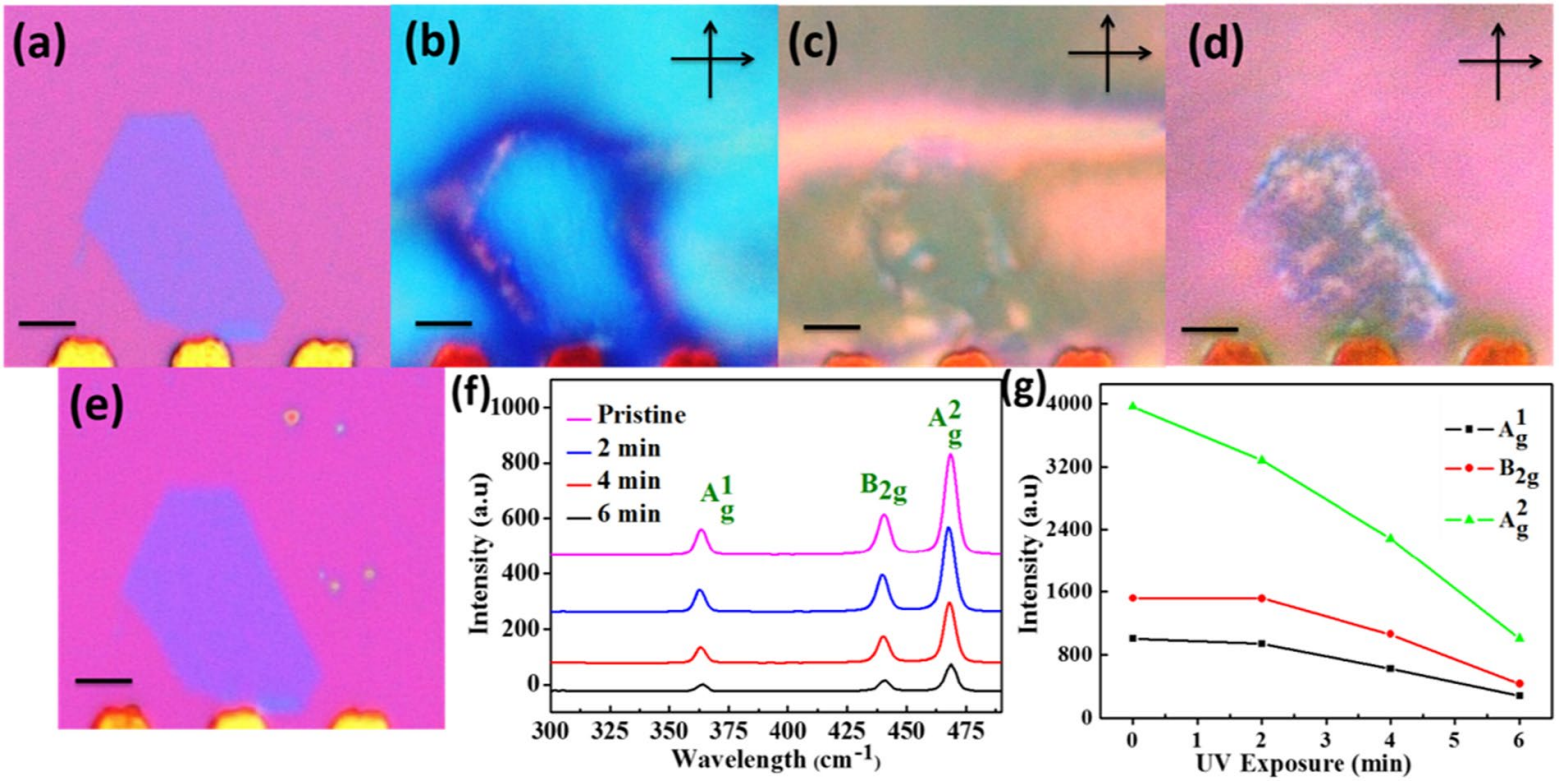
The LC alignment and the Raman analysis on the UV exposed BP flake. (**a**) The optical image of the BP before any exposure. (**b**–**d**) The LC alignment on the UV exposed flake for 2, 4 and 6 min. The increase in defect density is evident. (**e**) The optical image of the BP flake after UV exposures. (**f**,**e**) The Raman Spectra of the BP Flake with subsequent UV exposure and a Raman intensity plot, respectively. (**h**) The Raman Intensity plot of a relatively thicker flake. (scale bar = 5 µm).

Previous reports by Walia *et al*. established that exposure to the UV lights with different wavelengths for several minutes can significantly deteriorate the BP, and it can be confirmed by the AFM analysis. However, there aren’t any reports of the optical visualization of oxidation in the earlier stages. Furthermore, it was discovered by the experiments that the UV exposure for only a few minutes can oxidize the surface of the BP to an irreparable extent. However, oxidation induced by a shorter exposure to the UV light is not visible by the naked eye. To observe the defects induced by the UV on the BP, a thin flake of was exposed to a UV light in ambient for 2, 4 and 6 min. After each exposure samples were coated with the LC and then observed under a POM with a cross-polarizer (Fig. 3b–d.) The presence of the defects was clearly visible as shiny spots which emerged due to arise of new birefringence of the LC on the P_x_O_y_. With the UV exposure, the surface of the BP flakes get oxidized, which led to a change in the LC alignment along a new preferential orientation. It is interesting to note that no oxidation streak was observed after the removal of the LC (see Fig. 3e). No defects were visible in this image and it is the same as Fig. 3a, which is an optical image of the freshly cleaved flake. However, when the LC was coated on the same sample, the oxidation defects were clearly visible as shiny spots. This experiment further strenghthned our claim that the LC can be used to investigate and visualize the oxidation even in its earliest stage. It was also confirmed through the DFT calculation that the LC tended to align preferentially on the oxidized BP as compared to the pristine BP.

The Raman spectra were also collected from the pristine BP and after the UV exposure for 2, 4 and 6 min to study the defect formation via phonon vibration mode. All the intensities of the Raman active mode of the BP showed a decreasing trend with an increasing UV exposure, which is illustrated in Fig. 3f,g. This analysis was done on two flakes with different thicknesses, (see Fig. S1). We discovered that the decay in intensities is different for the different thicknesses of the flakes and it is sharper and more prominent for the thinner ones. This may be attributed to the faster kinetics of the thinner flakes as the UV exposure intervals were kept constant for both samples^11,12,14,30^.

**Figure 4:**
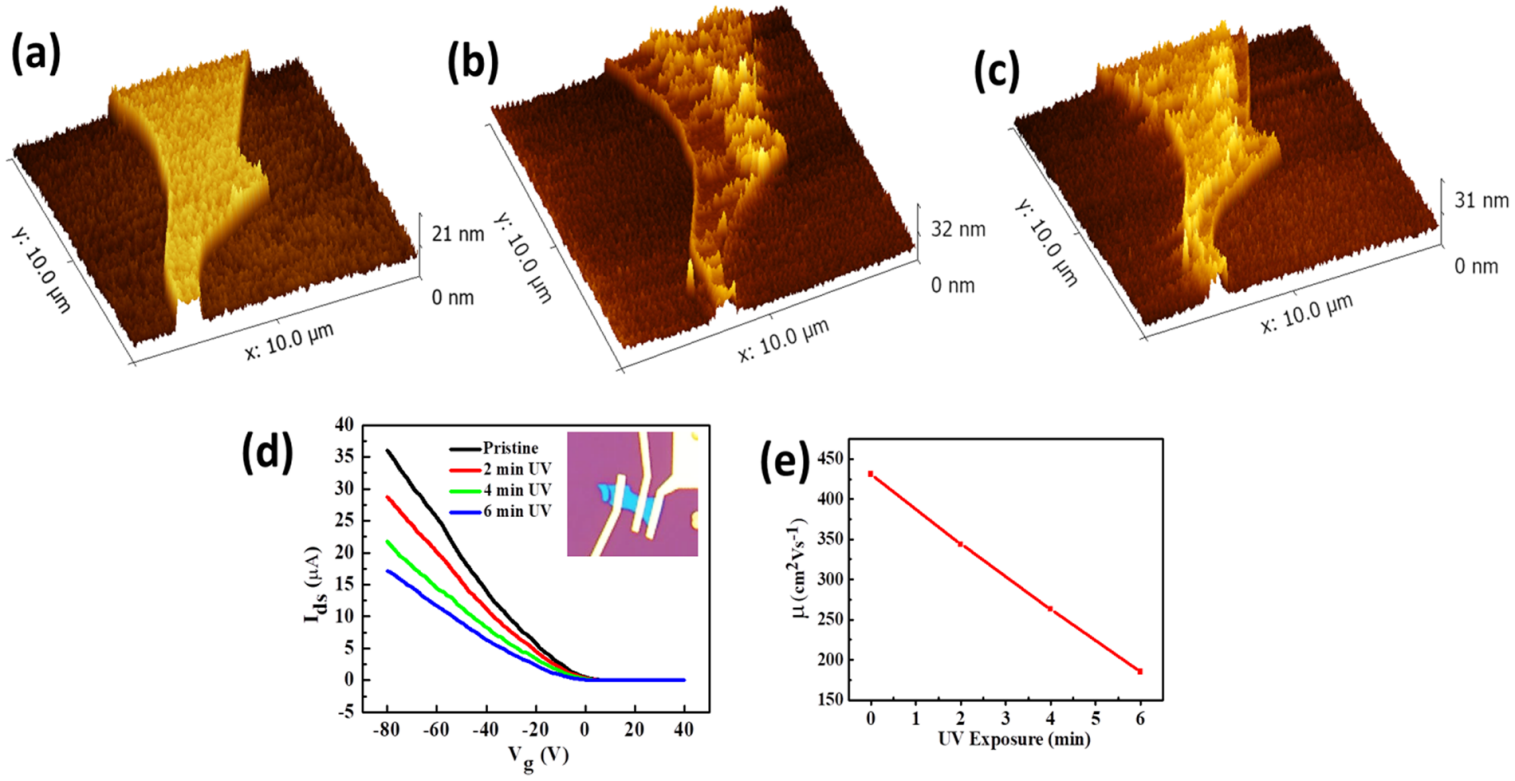
AFM topographical analysis and an electrical transport measurement with UV exposure. (**a**–**c**) AFM image of the pristine BP, after a 2 min UV exposure and a 4 min UV exposure respectively. The roughness has increased manifold with an increase in the UV exposure. However, some points show thinning, which can be attributed to the formation of the H_x_PO_y_. (**d**) The I_ds_-V_g_ curve of the pristine and the UV exposed BP. (**e**) The decreasing trend in mobility vs exposure time.

To gain further insight of the BP oxidation and to study the surface of UV oxidized BP, an AFM analysis was conducted. A freshly cleaved BP was subjected to an AFM topographical analysis which is shown in Fig. 4a. The surface of the pristine BP was relatively smoother and showed a height profile of 10 nm. This flake was exposed to UV exposure for 4 and 6 min and after each exposure the flakes were subjected to an AFM analysis. With each UV exposure, the surface roughness of the BP was increased which can be seen in Fig. 4b,c. The UV light increased the rate of oxidation of the BP and the surface roughness is the indication of ongoing oxidation. It can be seen that there was a major change in the topology of the BP. This change on the surface in-turn changed the alignment of the BP making it obvious with a visible light. As discussed earlier, the oxidation increased the resistance of the BP and thus deteriorated its electrical characteristics. In order to study the effects of the UV induced oxidation on the electrical characteristics, a FET device was fabricated. A BP flake was transferred on a PDMS stamp by micro-mechanical exfoliation using sticky tape, and the electrodes were patterned using e-beam lithography. About 5 nm Cr and 50 nm Au were thermally evaporated in a high vacuum chamber and deposited in the patterned electrodes. A BP FET was measured using a standard 2 probe measurement. Figure 4d shows the I_ds_-V_g_ curve for the BP FET. After measuring the pristine sample, the sample was subjected to UV exposure for 2, 4 and 6 min. It was noticed that there was a sharp decrease in the on current with an increase in the UV exposure time, which is shown in Fig. 4d. The charge carrier mobility was calculated for each curve, and we found a considerable decay in the charge carrier mobility with an increase in the UV exposure time, which is shown in Fig. 4e. Due to an increase in oxidation, charge trapping sites increased, which hindered the mobility of the charge carriers.

## Density Function Theory Calculations

**Figure 5:**
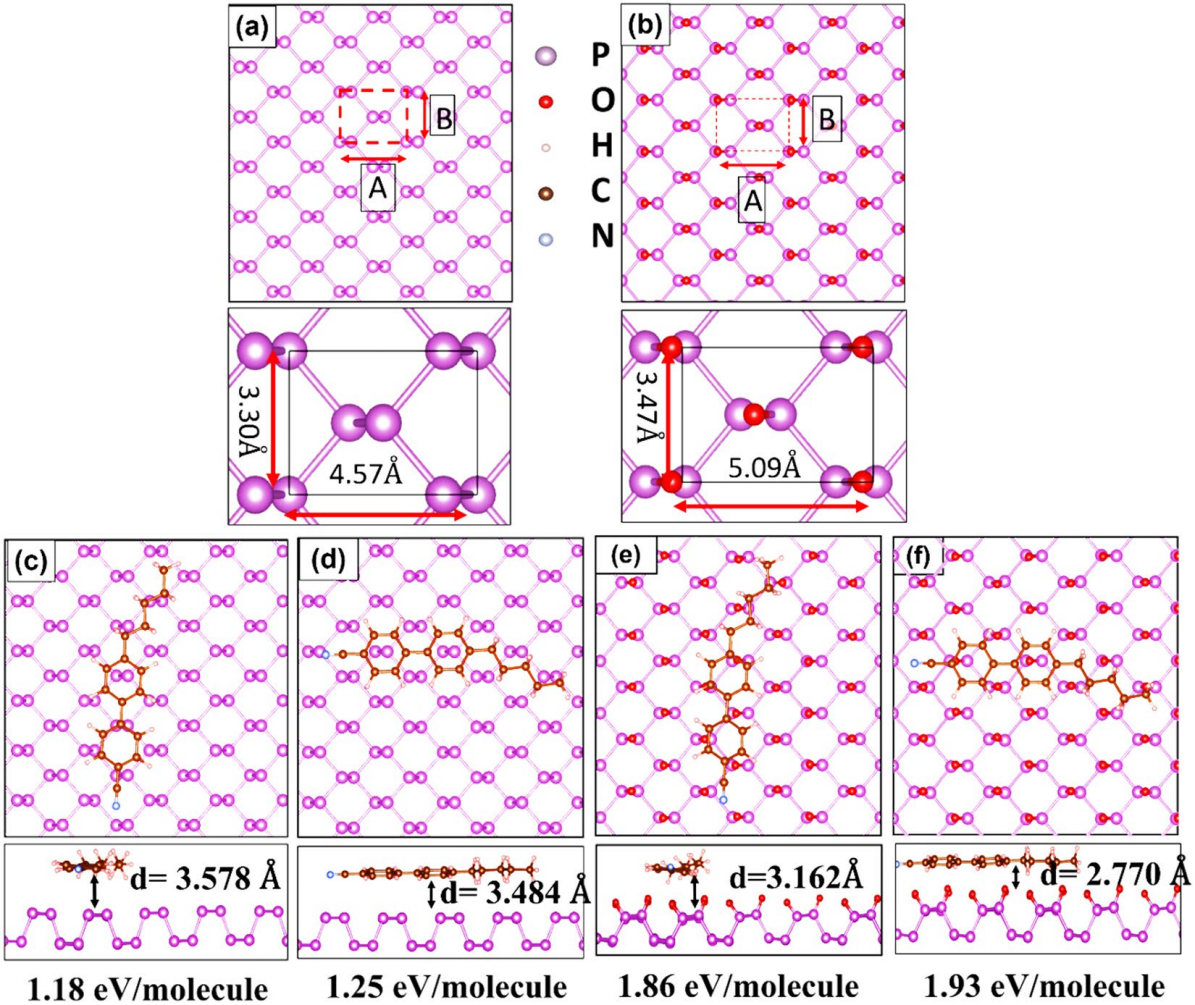
The DFT calculations result for a possible stacking configuration of the 5CB on the BP and the P_4_O_2_. (**a**,**b**) The top view of the BP crystal lattice and the P_4_O_2_. (**c**,**d**) The 5CB molecule aligned parallel to the zigzag and the armchair orientation on the BP, respectively, with adsorption energy and the distance between the centre of the LC and the BP lattice. (**e**,**f**) The 5CB align along the zigzag and the armchair orientation on the P_4_O_2_, respectively, with adsorption energy and the distance between the centre of the LC and the BP lattice.

From our studies, it is confirmed that we can optically visualize the BP and the degradation (oxidation) on the BP with the LC. As a result it was imperative to further gain in-depth knowledge about the LC alignment of the BP and the oxidized BP. To further extend our studies to the 5CB-BP system, the density functional theory (DFT) calculations are performed within a generalized gradient approximation (GGA) for the exchange-correlation (xc) functionals^31,32^, which were implemented in the Vienna ab initio simulation package (VASP)^33,34^. Figure 5a illustrates the calculated lattice of the BP and the unit cell is indicated by A and B lattice parameters. Due to the fact that the chemisorbed oxygen can form a number of bonds with the BP. Threrfore, different BP-O_2_ structures were considered (see Fig. S5). It was found that the P_4_O_2_ was the most stable energetically, as illustrated in Fig. 5b, and was considered further for our calculations. In this structure, one O atom is bonded to one P atom and due to the large electronegativity difference between the O and the P, which are 3.44 eV and 2.09 eV respectively, the P atom was dragged into the lattice^15^. Due to this, the lattice parameter along the A and the B direction varied with regards to the bond lengths in the pristine BP with a considerable differences of 0.12 Å and 0.17 Å respectively, which is illustrated in Fig. 5a,b. This variation could influence the anchoring of the LC molecules on the surface.

A favourable stacking configuration can be determined by adsorption energy which is defined as

$${{\rm{E}}}_{{\rm{ad}}.}={{\rm{E}}}_{5{\rm{CB}}}+{{\rm{E}}}_{{\rm{sub}}}-{{\rm{E}}}_{5{\rm{CB}}-{\rm{sub}}},$$

where E_5CB_ and E_sub_ is the energy of the LC-5CB molecule and the isolated the energy of the BP substrate, respectively. The E_5CB-sub_ is the total energy of the BP surface with 5 CB molecules adsorbed on it^19,35^. First, the LC preferential alignment was determined on the pristine BP. Due to an anisotropy in the BP crystal lattice, there was only a unique possible stacking of the BP along the zigzag direction which is illustrated in Fig. 5c. In this configuration, one bi-phenyl ring was attached in an AB style while the other one was in an A’A style. On the contrary, along the armchair atomic orientation 5CB molecule has the freedom to align in a AB and a AA stacking configuration. Based on the calculated adsorption energies value, the most favourable stacking mode was the AB style along the armchair direction. To compare the preferential alignments of the 5CB on the BP and the oxidized BP (P_4_O_2_), the binding energies of the 5CB on the P_4_O_2_ were calculated. Our calculations showed that out of all the considered systems, the LC alignment on the oxidized BP along a zig-zag and an armchair direction were more stable compared to its alignment on the bare phosphorous along the two same principal atomic orientations. These calculations further confirm our assertion that the LC has a tendency to align on oxidized BP, enabling us to visualize the oxidation.

## Conclusion

The BP is a highly unstable and readily oxidized in ambient conditions. The kinetics of the oxidation acts faster if it is exposed to UV light. The oxidation of the BP can’t be visualized optically but coating the LC on oxidized BP helps visualize the oxidation with the POM. It was observed that the birefringence property of the LC could be utilized to study the oxidation behaviour of the BP. The density function theory calculations confirmed that the oxidized BP could have a stronger binding with the LC compared to the bare BP, which helped to distinguish the oxidized and the unadorned BP surfaces. This study will help improve the passivation techniques of BP, and electronic properties of BP.

## The Experimental Details

### The fabrication of the BP-LC Cell

BP purchased from the 2D semiconductors, was exfoliated by the micromechanical cleavage technique using sticky tape and then it was transferred to a PDMS stamp. This cleaved BP was then transferred to a Si/SiO_2_ substrate with an oxide thickness of 300 nm. These flakes were used for the LC alignment and the degradation studies. The LC was coated using a spin coater at 2500 RPM for 40 s to acquire a film thickness of 0.2 μm. On the other hand, the PVA was coated on a glass slide at 3000 RPM for 30 sec. This cover glass was then rubbed in a specific direction and placed on the LC coated sample and samples. For the UV light exposure, a homemade setup was used, which was equipped with a UV discharge lamp irradiating a wavelength of 220 nm. After each UV light exposure samples were cleaned using acetone and methanol.

### Characterization Techniques

To conduct alignment study on the BP, the samples were observed with an Olympus BX-51 microscope that was fitted with a rotatable polarizer and an analyzer. The Raman spectroscopy was conducted using a Renishaw Raman apparatus with a laser wavelength of 514 nm and a spot size of 0.1 μm. The AFM was conducted in contact mode using a Park Systems AFM.

### Density Function Theory (DFT) Calculations

The DFT calculations were performed within a generalized gradient approximation (GGA) for the exchange-correlation (xc) functional^31,32^ which were implanted in the Vienna ab initio simulations package (VASP)^33,34^. The kinetic energy cut-off was set at 400 eV, and the electron-ion interactions were represented by the projector augmented wave (PAW) potentials^36,37^. Grimme’s DFT-D3 method^38^, which was based on the semi-empirical GGA-type theory was used for the Van der Waal correction. In the calculations, the LC was absorbed on a 5 × 8 unit cell of a monolayer BP and the oxidized BP. For the Brillouin-zone integration, we used a lxlxl grid for the atomic optimization of the absorbed 5CB on both the BP and the oxidized BP in the Gamma centred scheme. The atomic coordinates were fully optimized until the Hellman-Feyman forces became less than 0.01 eV/Å.

## Electronic supplementary material

Supporting materials
