# Supplementary material for: Visualizing Degradation of Black Phosphorus Using Liquid Crystals
Source: Sci Rep. 2018 Aug 28;8:12966. doi: 10.1038/s41598-018-31067-4 (PMC6113255; doi:10.1038/s41598-018-31067-4)
Supplement: Supplementary file 1 — Supporting materials [file 41598_2018_31067_MOESM1_ESM.docx]

**Visualizing Degradation of Black Phosphorus Using Liquid Crystals**

(Supplementary Information)

Bilal Abbas Naqvi ^1,2^ , Muhammad Arslan Shehzad ^1,2^, Janghwan Cha,^2,3^, Kyung Ah Min^2,3^, M. Farooq Khan^2,3^,Sajjad Hussain^1,2^, Yongho Seo^1,2^, Suklyun Hong ^1,2^, Jonghwa Eom^1,2^, Jongwan Jung^1,2*^.

^1^Departement of Nanotechnology &Advanced Materials Engineering, Sejong University, Seoul, South Korea

^2^Graphene Research Institute, Sejong University, Seoul, South Korea.

^3^Departement of Physics & Astronomy, Sejong University, Seoul, South Korea.

*Corresponding authors E-mail: [jwjung@sejong.ac.kr](mailto:jwjung@sejong.ac.kr) (Prof. Jongwan Jung)


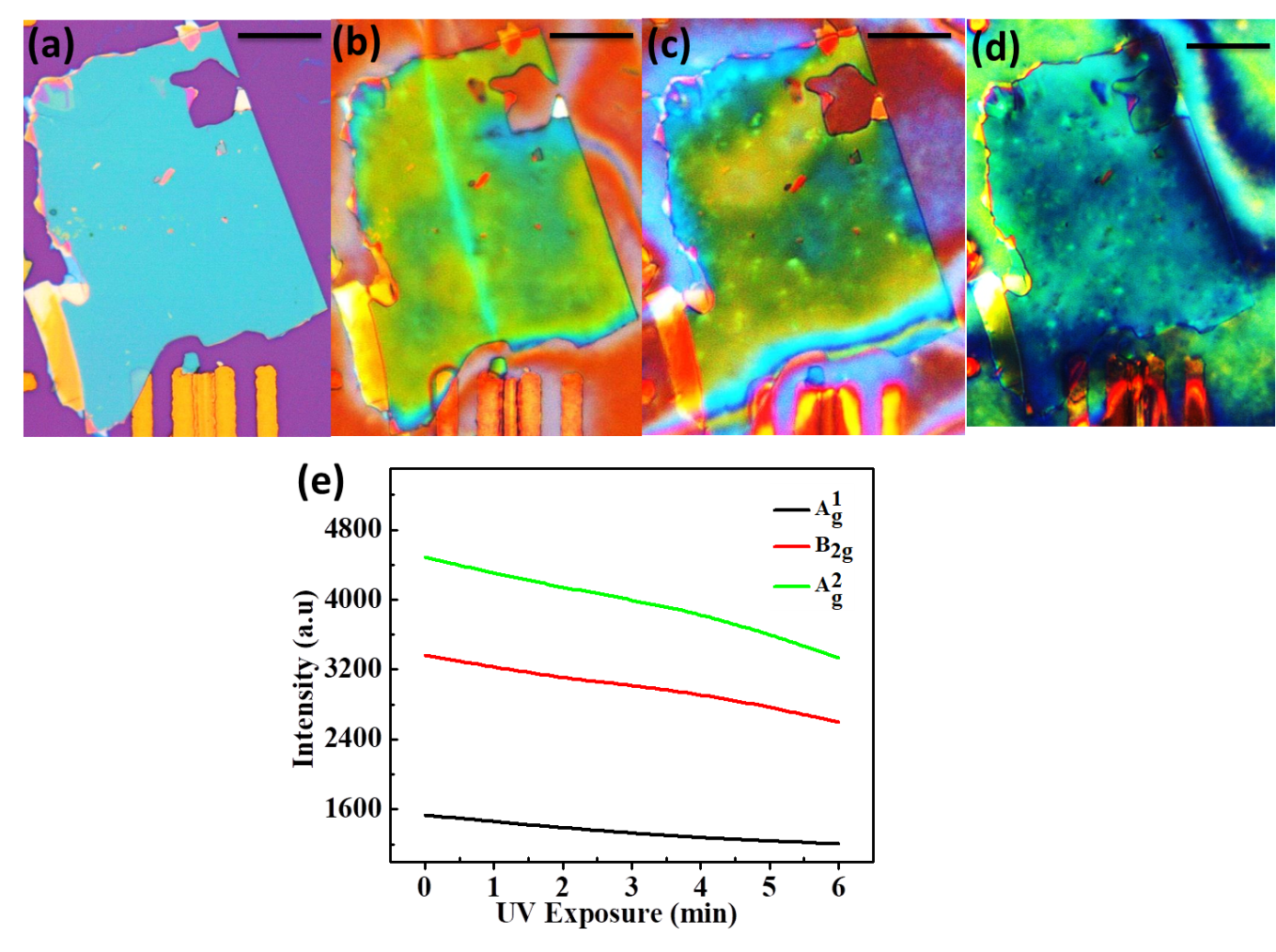


Figure S1. UV treatment of a relatively thicker flake and its effect on LC alignment on BP and Raman vibrational Mode Intensities. (a) Pristine BP with gold markers (b,c,d) POM image of LC Coated BP after subsequent UV treatment of 2,4 and 6 min respectively. There is an increase in defect density. (e) Raman intensity versus UV exposure time. (scale bar = 20μm)


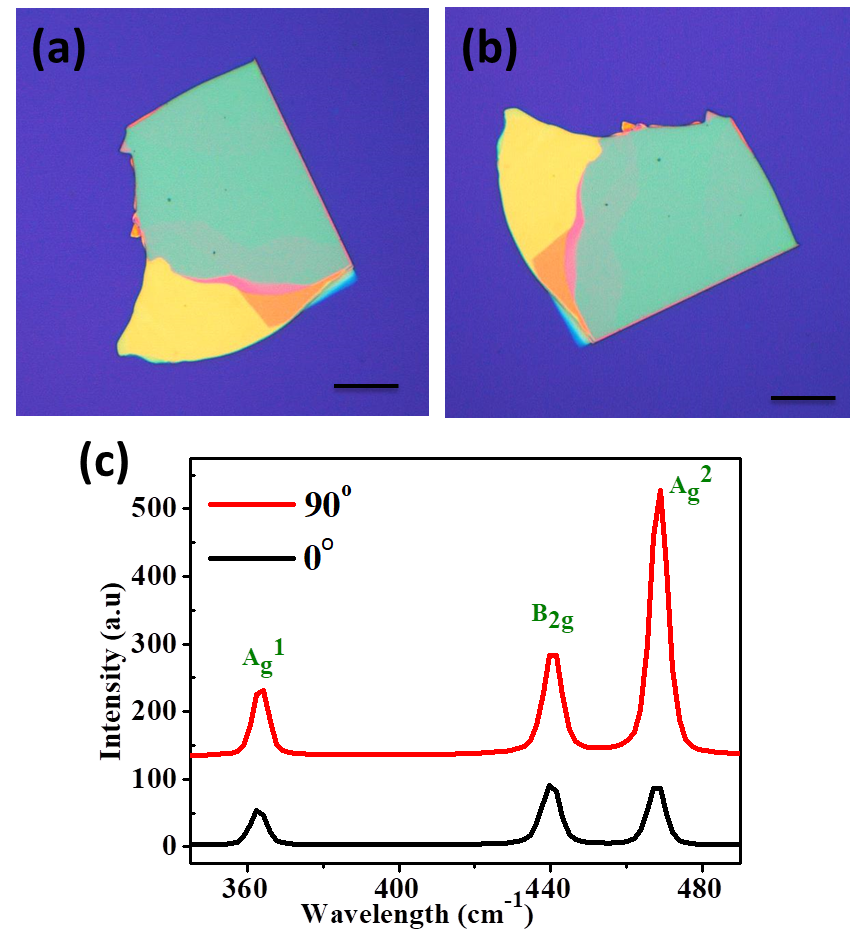


Figure S2. Angular dependence of Raman Modes of BP. (a,b) Optical image of freshly cleaved BP with 90 degree rotation. (c) Raman spectra of bp before and after 90^o^ rotation. There is sharp decrease in A_g_^2^ mode with 90 rotation which indicates that at 0^o^, the incident laser was polarized to zigzag atomic orientation and upon rotation to 90^0^ became parallel to armchair atomic orientation.


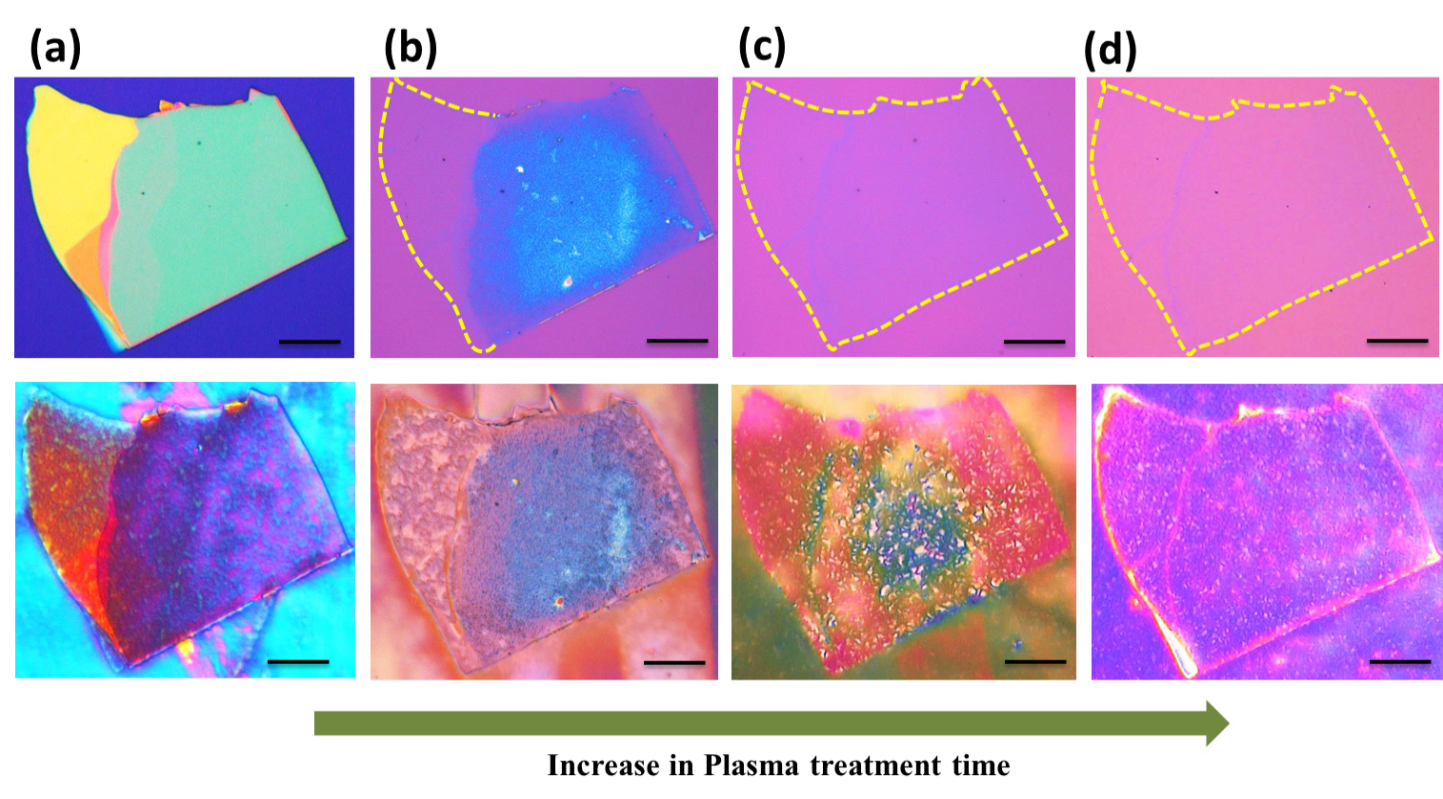


Figure S3. O_2_ Plasma treatment for and effect of LC alignment due to plasma treatment induced defects effect on LC alignment. (a) Optical Image of exfoliated BP and POM image of LC coated in lower frame. Creation of poly domains indicate presence of oxide on this BP (b,c,d) plasma treated flake for 5,10 and 15 sec shows thinning due to etching. Lower frame consist of images with LC coat after each plasma treatment. Eruption of poly domains is evident (scale bar = 10μm)


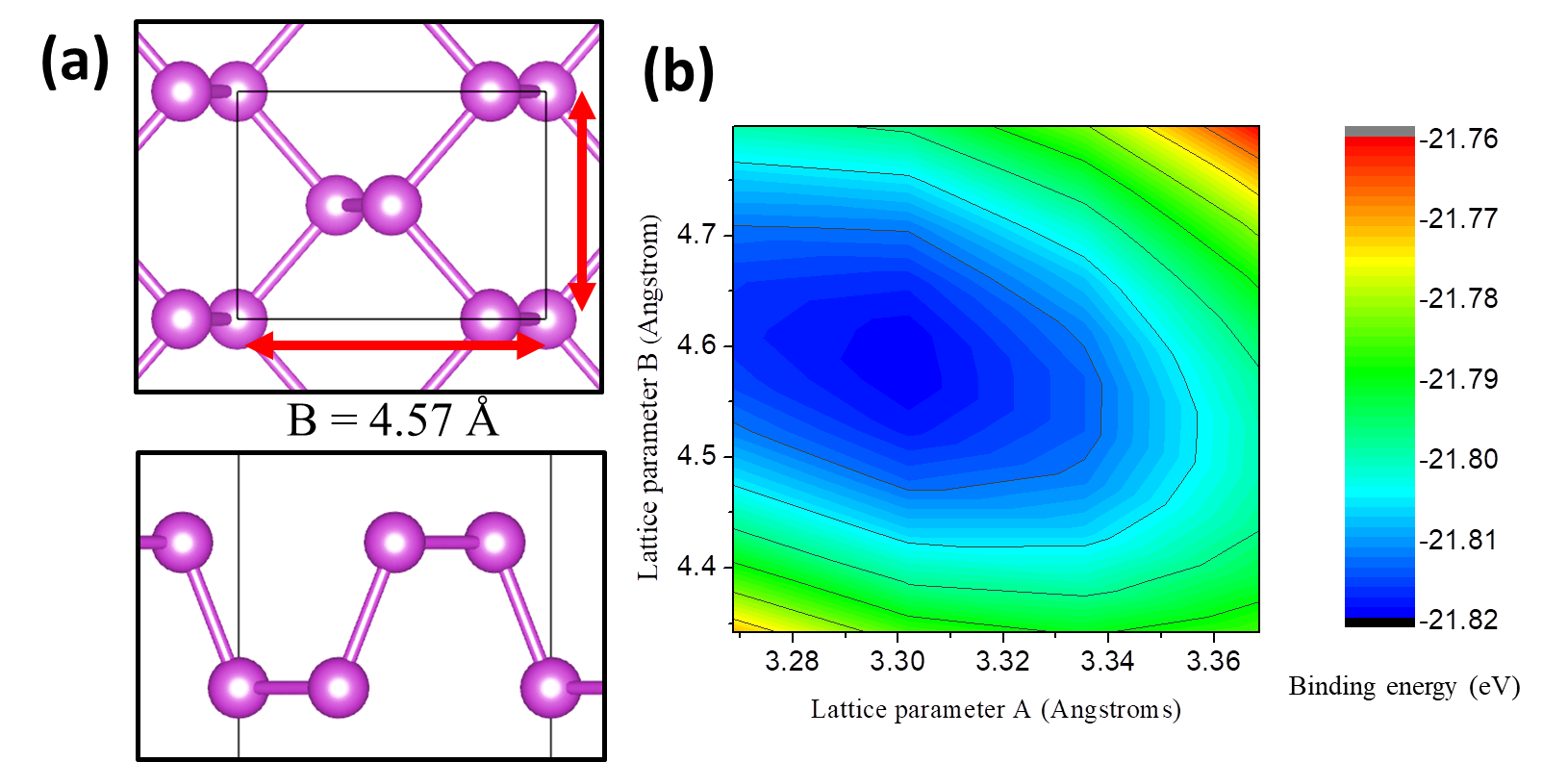


Figure S4. Calculated BP structure and binding energy per unit cell. (a) Top view and side view of unit cell of BP. (b) Binding energy against lattice parameter


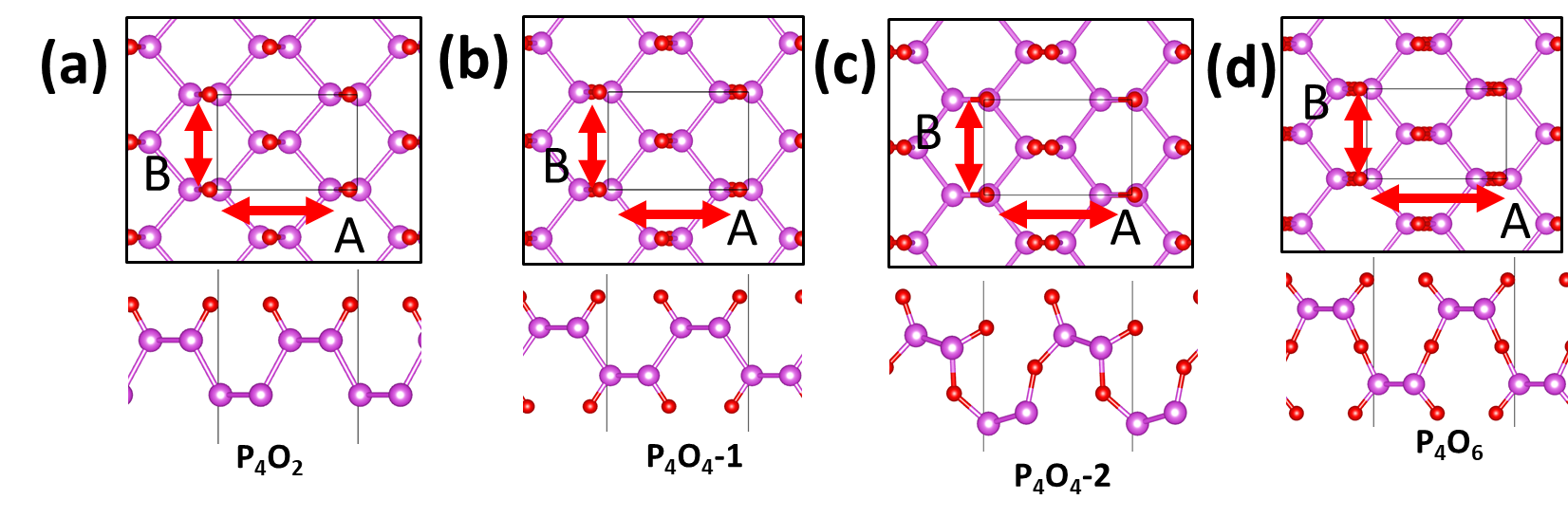


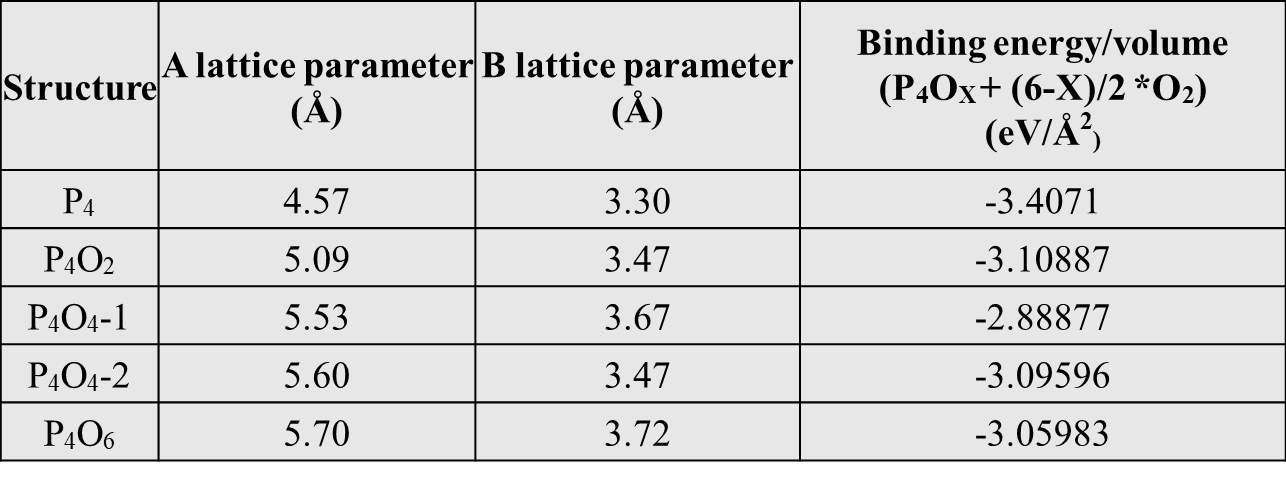


Figure S5. Calculated possible bonding configuration with phosphorus and oxygen. Table consists of lattice parameter and binding energy per volume.


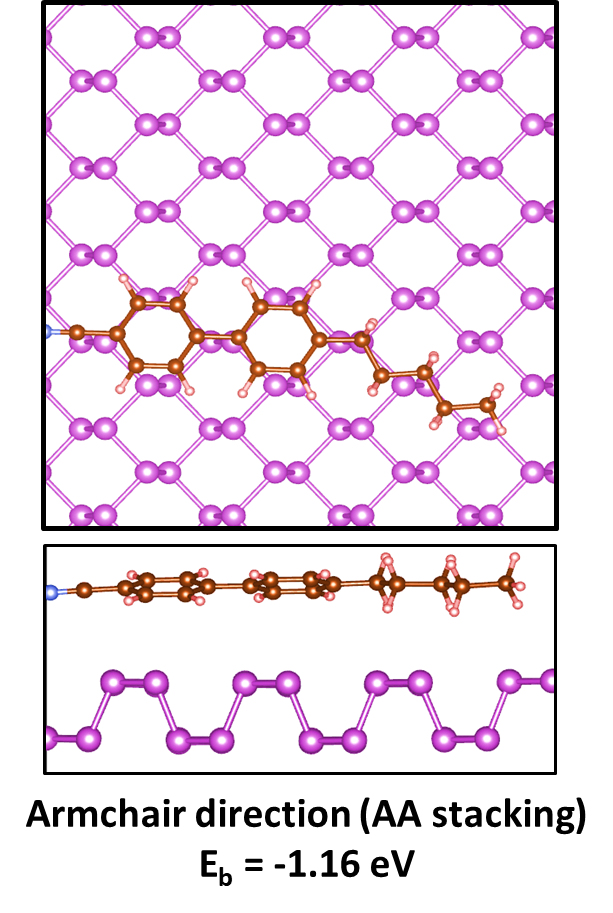


Figure S6. 5cb molecule stacked in AA configuration along armchair direction of pristine BP
